# The Journey to Comprehensibility: Court Forms as the First Barrier to Accessing Justice

**DOI:** 10.1007/s11196-021-09870-6

**Published:** 2021-11-15

**Authors:** Tatiana Grieshofer née Tkacukova, Matt Gee, Ralph Morton

**Affiliations:** Birmingham City University, Birmingham, UK

**Keywords:** Court forms, Comprehension, Written legal discourse, Litigants in person, Procedural justice, Noun phrases

## Abstract

The article explores the comprehensibility of court forms by providing a quantitative overview and a qualitative analysis of such syntactic characteristics as length and structure of sentences and noun phrases. The analysis is viewed in the broader context of genre characteristics of court forms, their role within legal proceedings, and their function for eliciting narratives from court users. The findings show that while the elicitation strategies are not always coherently aligned with the guidance sections, the guidance itself condenses legal and procedural information into overly complex and verbose syntactic constructions. Comprehensibility barriers are thus created through breaks in information flow, ambiguous syntactic constructions, missing information and misalignment between questions and guidance. Such comprehension challenges have a negative impact on the potential of court users to effectively engage with legal proceedings.

## Introduction: The Role of Court Forms in Legal Proceedings

In civil and family law, the first encounter of the court user with legal proceedings is often through a court form: either as the person completing a form to start the proceedings (i.e. claimant or applicant) or as the person responding to a court form (i.e. respondent). Court forms provide an opportunity for parties to present the preliminary information and set the ground for developing their case narrative; crucially, the micro narratives elicited in this way determine the administration and management of subsequent court hearings (e.g. whether the hearing would be conducted via a video link or in court). The process of completing a court form is therefore a key step in presenting the case to the judiciary, yet many lay court users experience multiple communication barriers due to the unawareness of court processes and procedures, insufficient knowledge of legal principles, or inexperience with legalese [50].

For the layperson, the effective engagement with court forms is conditioned by several successive cognitive and communication-related stages: (1) comprehension of the form and supporting documents (court form questions, guidance accompanying the form, text in the intertextual links); (2) successful semantic mapping (identifying semantic features of the relevant legal concepts, reducing semantic distance between the legal meaning and lay understanding of the concepts, framing the case arguments within the pertinent semantic field of applicable legal concepts); (3) communication of the case narrative through close-ended and open questions in the court form (framing the narrative within legally coherent boundaries in a genre-specific manner, including numbered paragraphs, statement of truth and other genre-specific requirements).

The article focuses predominantly on challenges related to the first stage because comprehension is a pre-requisite for the successive stages of completing a court form. The study provides a corpus-based overview of the lexico-grammatical complexity, which impacts the comprehension of civil and family law forms used in England and Wales. By exploring two sub-corpora, one with 35 most frequently downloaded court forms used in civil and family proceedings and the other one with 50 court forms used in child-related private and public family proceedings, the study illustrates the scale of linguistic barriers lay court users most frequently encounter either when pursuing most common claims and grievances or when trying to make child arrangements.

The study was motivated by several changes which have impacted the way courts have been administering and managing cases in England and Wales in the last two decades. The first change was introduced by the drastic cuts in legal aid as a result of the Legal Aid, Sentencing and Punishment of Offenders Act 2012. Reducing the availability of legal aid shifted the responsibility for accessing justice from the state to the individual, without the provision of a support structure for those who cannot afford a legal representative [28]; this caused the gradual increase in numbers of litigants in person (or self-represented litigants—people who represent themselves in court proceedings). For instance, in private family proceedings, the number of hearings where at least one party is unrepresented has increased from 55% in 2012 to 80% in 2020.^1^ As a result, the judiciary are often presiding over the hearings where at least one of the parties is not represented. Accordingly, the number of court users who are completing forms without the support of a lawyer has also increased dramatically. And although there are charities (e.g. Support through Court) which offer support with filling in court forms and completing paperwork for court, many self-represented litigants are either not aware of the services [26] or struggle to reach them due to their availability or location [28]. The high number of unrepresented litigants provides a sufficient rationale for enhancing the comprehensibility of court forms. Represented clients would also benefit from the changes because even if they do not complete the form themselves, they often need to check the form is correct and represents their narrative accurately before signing the statement of truth.

The second reason for exploring the comprehensibility of court forms is given by the move towards digitization of the justice system introduced as part of the HMCTS (Her Majesty’s Courts and Tribunals Service) reform. Innovations, such as online applications and virtual courtrooms, were being developed as part of the reform programme and their use was further accelerated by the COVID-19 pandemic.^2^ In an online environment, comprehension is key to making any progress with a court application because remote settings mean that there may not be immediate support available and court users need to concurrently manage technical, communicative, procedural and legal aspects. Interestingly, online applications seem to be viewed positively by court users. The digitized version of the C100 form used for child arrangements reported 92% user satisfaction, but it is important to bear in mind that the measure only reflects online user experience (webinar presentation at *4th HMCTS Annual Public User Event*, November 2020) rather than the success rate of applications or the degree to which court users understood the instructions and managed to present their narrative in a legally coherent manner. There is an attempt within HMCTS to highlight the role of clear communication through projects which aim to provide guidelines for achieving a friendly tone when communicating with court users (e.g. the Human Voice of Justice), but these attempts have not reached the court forms yet. The digitization of court forms presents an opportunity to redesign the forms, including elicitation processes embedded within them and language use in guidance sections or supplemental documents.

The third reason for focusing on comprehensibility of court forms lies in the fact that courts are hesitant to give self-represented litigants any leeway if the guidance is accessible and reasonably comprehensible. For instance, in the case of Barton v Wright Hassall, it was decided in a 3–2 decision in the Supreme Court in 2018 that despite the difficulties of acting in person, the availability of the guidance (rules and practice directions) online should be sufficient:

Unless the rules and practice directions are particularly inaccessible or obscure, it is reasonable to expect a litigant in person to familiarise himself with the rules which apply to any step which he is about to take.

A key assumption of this ruling is that rules and practice directions for navigating the legal process are not inaccessible or obscure to someone without a professional legal background. Yet relatively little work has been done to examine the accessibility and comprehensibility of procedural rules and directions for lay court users. From the linguistic point of view, there is potentially an even stronger argument in favour of examining the comprehensibility of court forms as they act as a legal-lay communication tool [51]; any barriers to their comprehensibility would thus have a direct impact on access to justice.

In circumstances when court forms are often being completed by self-represented litigants without much support with legal, procedural, digital or communicative aspects, the questions around their comprehensibility have never been so pertinent for accessing justice by the most vulnerable. By providing a quantitative overview of linguistic features in the corpus of civil and family court forms, the article explores lexico-grammatical and syntactic characteristics which have been shown to reduce accessibility and impede on comprehension (e.g. nominalisations) and reflects on the challenges self-represented litigants experience when presenting their initial narratives to the judiciary through court forms.

## Existing Research on Comprehensibility of Legal Discourse

The comprehensibility of court forms has so far been explored by a limited number of studies [33, 38, 40, 51], which generally concluded that substantial changes are required to simplify language use and improve the understanding of court forms among the wider population [33, 51] or that due to their complexity, court forms should not be used without legal support [38]. Broader issues on the comprehensibility of written legal texts have, nonetheless, been researched in more depth through several methodological lenses. While language and the law scholars have mostly focused on genre characteristics and linguistic features of legal texts [8, 10, 24, 45–48] or contributed to the exploration of drafting and interpretation processes [5, 35, 42, 43], applied psychologists have been focusing on experimental studies measuring the degree of comprehensibility of legal texts in various settings [19, 30, 33]. This study draws on both strands, but methodologically follows in the footsteps of the lexico-grammatical approach because of the existing gap in our understanding of court forms as a linguistic genre; the genre characteristics of court forms, their role in narrative elicitation, and their function within legal proceedings need to be better understood before exploring them from an experimental or empirical perspectives.

The linguistic characteristics of written legal discourse reflect the complex and decontextualized nature of legislation: long sentences, complex sub-ordination of clauses within sentences, syntactic discontinuity, complex nominal structure, multi-nominal expressions, polysemy of legal terminology, minimal punctuation use [7: ch. 5, 11: 106, 14, 22, 32]. The linguistic complexity is a result of the need to ensure the legal scope and legislative intention are clearly expressed during the drafting process [12]. But the requirement for all-inclusiveness and at the same time for precision of expression and unambiguity of interpretation comes, partially, at the cost of clarity and transparency [12, 18: 162–169, 44]. Finding the middle ground between clarity and transparency, on the one hand, and all-inclusiveness and accuracy, on the other hand, has become the holy grail of research on legislative drafting, legislative interpretation and intelligibility and comprehensibility of written legal discourse.

The Plain Language Movement and the discussion on plain language principles have provided one perspective for addressing the challenge. Established in the 1970s, first in English-speaking countries and then more internationally, the movement and the associated campaigns (including the UK-based Plain English Campaign) have contributed to the shift in drafting and move away from legalese to easified texts [7]; the simplification process typically includes the use of syntactically simpler and shorter sentences, concrete language (as opposed to abstract language), explanations of terminology and concepts. The plain language principles also promote the recognition of the importance of clear design and organisational structure in aiding comprehension [1]. The experimental studies comparing written legal texts of different genres to their simplified versions show that such changes tend to have a positive impact on the readers’ comprehension [33], but do not reduce all the comprehension barriers. The barriers which tend to persevere are linked to discursive, cognitive, and extra-linguistic challenges, which are not easy to address. For instance, Masson and Waldorn’s [30] experimental study shows that beyond the simplification of the micro-linguistic features, it is also important to explore macro-linguistic features, such as simplifying discourse-level characteristics, clarifying the intention of the text and strengthening the coherence of the propositional content. Explicitly explained propositional content helps the lay user apply legal concepts in practice and explain their reasoning, and thus engage in professional discursive activities [19]. In addition to explanations, Lieberman [27] highlights the positive impact visualisations and illustrations of legal concepts have on improving the overall level of comprehensibility.

The cognitive and extra-linguistic barriers are given by the discrepancies in the legal and lay knowledge schemata and variations within socio-cultural perceptions, which can be difficult to address. The differences between legal and lay knowledge schemata, whether they have common areas of overlap or not, impede on the comprehension process; lack of understanding or lived-in misconceptions about law thus curb the effectiveness of court users’ participation in legal proceedings [30, 34]. Even after the explanation or clarification of common misunderstandings, the misconceptions do not seem to disappear [30]. Further socio-cultural barriers are created through the pervasive presence of unequal power relations in legal settings, manifested via the exclusiveness of legal discourse [12, 18] and the hesitancy of the legal profession to include lay court users as more equal participants (e.g. it is much more difficult for litigants in person to instruct expert witnesses due to court procedure rules and/or the language of instructions and communication strategies expected in legal settings [50, 52]). This, naturally, influences the layperson’s engagement with written/spoken legal discourse, and at the same time impacts the way legal professionals make decisions on which content to include or excluded in the guidance documents: (lack of) transparency can be used to create more powerful positions [12].

The inherent meaning, as opposed to explicitly expressed content, creates an additional barrier for the layperson’s comprehension of legal texts [3]. Assy [2] argues that understanding legal discourse involves the awareness and understanding of the legal concepts, rules, doctrines and principles as well as the ability to categorise these accordingly, apply them to a given situation and create coherent legal arguments; this requires comprehensive legal training and cannot be achieved just by reading a plain version text. Ződi [56: 246] highlights that it is not the language or syntactic characteristics of legal documents, but “the systemic and interpretative character of law and the growing importance of technical rules that hinder the understanding of legal texts”. The complex and interwoven character of the legal system and the fact that many pertinent links are often not explicitly expressed in legal texts make it more difficult to distinguish between legal and procedural information. Yet, this distinction is crucial for guidance materials as blurry boundaries between the two types of information sometimes result in procedural guidance being deemed as unsolicited legal advice and thus being excluded from guidance materials [36]. Procedural information is, however, as important as legal information [52: 83]. Public legal education is one way of addressing common misconceptions and introducing improved understanding of court procedures [18: 199, 30], but the other crucial component is the provision of tailored legal and procedural advice and guidance materials [38, 49].

Without any form of support, completing a court form can be a daunting experience due to the above-mentioned challenges, ranging from complex lexical choices and syntactic constructions to implicit legal and procedural systemic links. Despite the strong sentiment that linguistic simplifications are not sufficient to achieve a reasonable degree of comprehension of legal texts among the wider public, it is crucial to explore lexico-grammatical and syntactic characteristics of court forms because they function as the initial remote interaction tool between legal professionals and lay court users. Moreover, court forms embed several communicative functions, such as eliciting information, providing guidance, and giving instructions on how to complete questions [51]; yet, there is a gap in understanding court forms as a genre or their role in eliciting information. The linguistic complexity of court forms is currently not fully appreciated and the redrafting of court forms has not always been successful in the past; the digitisation of court applications will, nonetheless, create the need for further redrafting, hence the research into their lexico-grammatical characteristics is timely. For instance, Tkacukova [50] discusses the first sentence from the form A (a private family law application for making post-separation financial provision): after several revisions of the form, even the very first sentence contained many comprehension challenges (grammatically complex language, double negation, dual conditions, ambiguous syntactic structure, lack of guidance on how to fill in the form. In the newly redrafted version,^3^ many of these challenges still appear (complex vocabulary and complex sentences with multiple embedded clauses). A more recent study on court forms [51], comparing the downloadable pdf version of the court form C100 (used for making arrangements for a child or resolving a dispute about their upbringing) to its online interactive version shows that the guidance is often insufficiently presented, the elicitation strategies embedded in court forms do not support the narrative construction, and legal concepts are not framed in a clear manner.

There is thus a strong rationale for exploring the lexico-grammatical and syntactic characteristics of court forms. The focus in this article is on the longer sentences and noun phrases because they tend to condense a lot of information [31, 49]. It is important to note that the length of sentences and syntactic constructions does not necessarily directly link to the level of complexity as longer syntactic constructions can be more explicit and transparent than shorter sentences or noun phrases [15]. The focus on the long syntactic constructions in this article is justified by the hybrid genre characteristics of court forms and more diverse functional requirements on longer constructions (see below).

## Data and Methodology

The study draws on two datasets: sub-corpus 1 with 35 most frequently downloaded forms from the HMCTS website gov.uk (these were identified through personal email communication with HMCTS staff) and sub-corpus 2 with 50 court forms related to child arrangements as part of private and public family law proceedings. While the first sub-corpus aims to provide an overview of the court forms used by a wide range of court users party to civil and family proceedings, the second dataset creates a lexically unified sub-corpus centred around the legal concepts and court processes relevant to private and public child-related family proceedings.^4^ The rationale for the two datasets is driven by the following factors: the balance between the wide range of the topics (sub-corpus 1) and the focused approach on one type of cases (sub-corpus 2); the balance between reflecting the experience of the wider court user base (represented clients and self-represented litigants) and the experience of court users in family proceedings where self-representation is more predominant; the the upcoming digitisation of court forms for private and public family proceedings as part of the HMCTS reform (the first form, C100, was digitised in autumn 2020 and further forms should be available online during 2021–2022).

The court forms were downloaded from the official court finder website (gov.uk) in their original format (mostly pdf format and a few forms in the doc format). In order to prepare the files for the corpus, it was necessary to extract some sections (e.g. tick boxes for close-ended questions or empty boxes designed for open questions) or reformat sections which were incorporated as images and were thus not machine-readable (such text was copied or transcribed). Due to the genre-specific characteristics of court forms and their function of eliciting information and guiding court users, the files contain a mixture of full sentences in the form of declaratives (‘*Please specify the nature of the order you seek*’, form C100) and interrogatives (‘*What order(s) are you applying for?*’, form C100) as well as incomplete sentences, especially noun phrases (‘*Prohibited Steps Order’* or ‘*evidence of a relevant police caution for a domestic violence offence*’, form C100) and subordinate clauses (‘*If the child arrangements order is not being complied with’ or ‘to revoke an existing enforcement order*’, form C79).

**Fig. 1﻿:**
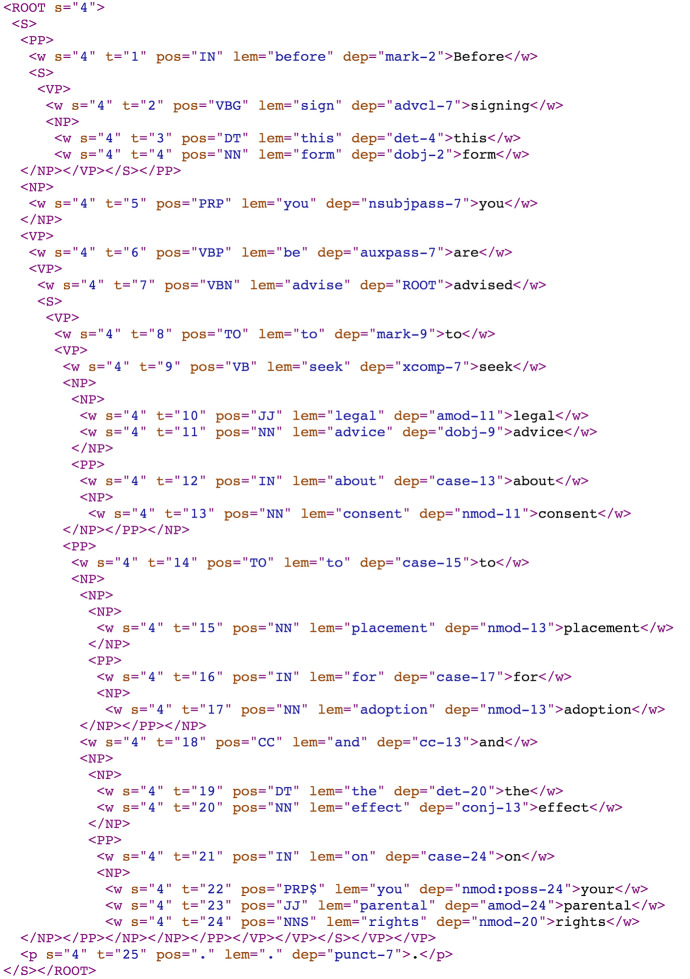
An example sentence (“Before signing this form you are advised to seek legal advice about consent to placement for adoption and the effect on your parental rights.”) in XML format following constituency and dependency parsing. Closing XML tags (starting with < /) are grouped onto single lines for brevity

The study presents a corpus-driven lexico-grammatical and syntactic analyses of the two sub-corpora. The quantitative information was extracted from the text using sentence parsers, specifically the constituency and dependency parsers from the Standford Core NLP suite of tools [29] and the CQL concordance function in Sketch Engine^5^ to extract verb phrases in passive voice or search for different structural combinations within noun phrases. The parser results were saved in XML format; an example sentence is shown in Fig. 1. The constituency parser allowed the identification of noun phrases and other sentence components, represented by the XML tags < *S* > (sentence or clause)*,* < *NP* > (noun phrase), < *PP* > (prepositional phrase*)* and < *VP* > (verb phrase). Words are shown within < *w* > tags (and punctuation within < *p* > tags) with attributes for sentence number (*s*), sentence position (*t*), part-of-speech (*pos*), lemma of the word (*lem*) and sentence dependency (*dep*).

The hierarchy of the XML tags represents the hierarchy of the constituency parse. For example, *Before signing this form* (Fig. 1) was evaluated by the constituency parser as a prepositional phrase (< *PP* > *;* starting with *Before*) which contains a clause (< *S* >) with a verb phrase (< *VP* > ; with root *signing*) and a noun phrase (< *NP* > ; *this form*). This information was used to investigate the complexity of the phrases and sentences in terms of numbers of embedded phrases (phrases within phrases), sentence length (number of words) and sentence fragmentation (sentences missing a verb). The XML format files were searched using XQuery (for an overview see [39]) in the BaseX software^6^ to create statistical summaries of the frequency, average length, and average number of embedded elements of the noun phrases (specifically, embedded noun phrases, prepositional phrases, parentheticals and sub-clauses).

**Fig. 2﻿:**
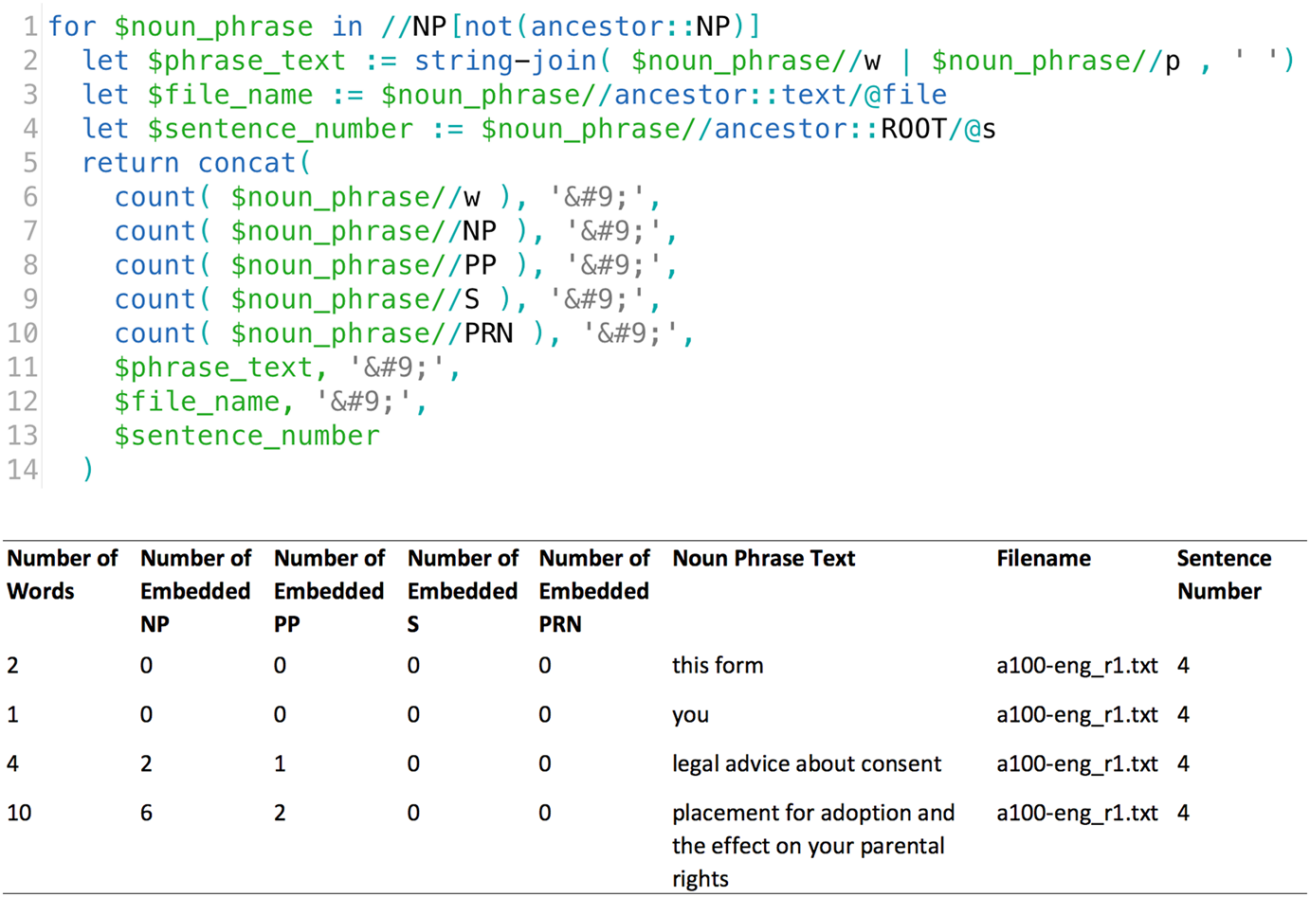
(Top) XQuery to extract counts of phrases and sentence components contained within noun phrases. (Bottom) Results from the example sentence in Fig. 1

Figure 2 shows the XQuery used to extract information about noun phrase complexity from the XML files. The first line of the code identifies the ‘top-level’ noun phrases (i.e. noun phrases that are not contained within another noun phrase). A double slash (*//*) searches within the XML hierarchy for child nodes matching the specified XML element. Lines 6 to 10 search for and count the words (*//w*), noun phrases (*//NP*), prepositional phrases (*//PP*), sub-clauses (*//S*) and parentheticals (*//PRN*) contained within each top-level noun phrases. The other lines concern formatting of the results and extract useful supporting information, such as filename and sentence number. The example sentence shows that the software counted four top level noun phrases: *this form, you, legal advice about consent, placement for adoption and the effect on your parental rights*. The last two phrases, in fact, belong to the same noun phrase, but were separated by the parser because of the nominal verb *consent*. Mistakes of this type cannot be prevented due to the amount of data analysed and the imperfect accuracy of even state-of-the-art parsers, but they are accounted for during the discussion of the findings. The figure also shows that there are three prepositional phrases embedded in the noun phrases (i.e. prepositional phrases starting with *about, for, on*) and eight embedded noun phrases (*legal advice, consent, placement for adoption, placement, adoption, the effect on your parental rights, the effect, your parental rights*). The information extracted from the parsed texts and CQL searches in Sketch Engine was used to generate the summary statistics presented in Tables 1–2.

**Table 1:** Corpus information – number of court forms, words, complete sentences, incomplete sentences, verb phrases in passive voice, noun phrases

	Sub-corpus 1 (most frequent forms)			Sub-corpus 2 (child related forms)		
	Total	Average	Standard deviation	Total	Average	Standard deviation
Number of court forms	35	–	–	50	–	–
Number of words	113,645	3,247	4268.18	42,767	855.34	1129.14
Number of complete sentences	5,081 (55.5% of syntactic constructions)	145.17	182.64	1,876 (43% of syntactic constructions)	37.52	47.46
Length of complete sentences	–	15.00	4.03	–	15.58	5.09
Number of incomplete sentences	4,067 (44.5% of syntactic constructions)	116.2	109.29	2,451 (57% of syntactic constructions)	49.02	58.97
Length of incomplete sentences	–	5.85	2.59	–	6.20	2.25
Number of Verb phrases in passive voice	1389 (1.2% of the sub-corpus)	39.68571	50.58352	704 (1.6% of the sub-corpus)	14.08	14.24543
Number of Noun phrases	20,324 (17.9% of the sub-corpus)	580.69	736.73	7,815 (18.3% of the sub-corpus)	156.30	192.14

**Table 2:** Structure of Noun Phrases in the sub-corpora

	Most frequent forms		Child related forms	
	Average	Standard deviation	Average	Standard deviation
Length of Noun Phrases (in words)	4.14	0.88	4.24	1.02
Number of Embedded Noun Phrases	1.34	0.48	1.52	0.65
Number of Embedded Prepositional Phrases	0.39	0.15	0.43	0.14
Number of Embedded Clauses	0.17	0.08	0.16	0.13
Number of Embedded Parentheticals	0.06	0.05	0.13	0.11

## Beyond the Syntactic Complexity

This section presents the sub-corpora and the linguistic features associated with reduced comprehensibility, such as sentence length, noun phrases and passive voice. Table 1 provides a quantitative overview, which is then followed by the qualitative analysis and discussion of genre characteristics of court forms and the impact of the syntactic structure of sentences and incomplete sentences (phrases or subordinate clauses occurring independently) on comprehensibility and access to information.

The high degree of deviation throughout different categories points to differences among the court forms. The deviation is generally lower in the sub-corpus 2 with child-related forms because these forms are managed by the HMCTS teams on private and public family proceedings and thus share the micro institutional discourse within HMCTS and similar legal domains. Since sub-corpus 1 covers a broader spectrum of court forms, the higher deviation shows more variety among the individual legal areas and the broader spectrum of institutional discursive cultures within HMCTS.

Overall, the average length of syntactical units in both types of sub-corpora is 15 words per sentence and around six words per phrases or clauses functioning as incomplete sentences, but there is a lot of variation not only between the sub-corpora or among individual forms but also within the forms. For instance, for some forms the average length of sentences is 27.53 words (‘A101: Consent to the placement of my child for adoption with identified prospective adopters’) with the deviation as high as 10.61. Interestingly, even the form A101 with the highest average sentence length does not reach the average estimated sentence length for legal discourse; the length of sentences in legislative documents has been estimated as follows: 55.11 words per sentence in *Courts Act 1971* [20], 45.05 words in a sample of statutes [23] or 37.06 words in the statutes collected in 1990 [25]. Legislative documents form a clearly defined genre with specific drafting rules to conform to [9], which generally require longer sentences. There are thus two reasons for the lower average sentence length in the analysed court forms.

Firstly, the software methods used here were only able to identify sentences which were presented in a linear way; the graphically separated sentences with several options to choose from were treated as syntactic segments (e.g. Example 3). This potentially skews the overall picture of such quantifiable characteristics as the overall length of sentences (the average length of sentences would, in practice, be higher) or the ratio between sentences and syntactic segments (Table 1 shows that sentences amount to 55.5% and 43% of syntactic constructions in the analysed sub-corpora, but in practice the ratio of sentences would be higher). Due to the amount of the text analysed and the frequent use of non-linear sentences in legal discourse [22], it was not possible to manually amend tagging of individual sections for their syntactic characteristics.

Secondly, court forms are a heterogeneous genre: they have several diverse communicative aims (elicit information, define legal scope and provide guidance on how to complete questions) and include diverse syntactic constructions and functions (interrogatives, imperatives, declaratives, independent phrases, independent subordinate clauses). The explanatory sections intertwine with information-eliciting sections, which leads to discontinuity in information flow and breaks in elicitation strategies. The forms thus combine different genres and incorporate guidance with information elicitation: instructions on what to include, explanations of legal concepts and multiple intertextual links often followed/preceded by individual questions [51]. The sentence average as presented in Table 1 thus does not reflect the syntactic complexity within individual forms (e.g. information-eliciting sections vs. guidance sections); but due to the quantity of data and the close integration of the guidance and information-eliciting sections, it was not possible to tag sentences for their functions.

To address the above mentioned challenges and to explore the syntactic complexity of forms in more depth, we qualitatively examined (1) the top 50 longest and shortest sentences and fragments across the sub-corpora and (2) all syntactic constructions in one court form shared between the sub-corpora (‘C100: Apply for a court order to make arrangements for a child or resolve a dispute about their upbringing’) in order to contextualise the length and function of sentences (see a more detailed analysis of the C100 form in [51]). As discussed elsewhere [51], shorter sentences and syntactic segments are mostly part of sections eliciting everyday information (e.g. personal details) or specific legal information (e.g. which court order the applicant is applying for), or provide information on procedures (e.g. confidentiality of information) and thus tend to refer to concepts which are familiar from workplace and general administration domains. The comprehension challenges they cause are linked to the use of legal concepts or legal procedures [49]. Longer sentences include more propositional content expressed through complex lexico-grammatical constructions [51]. Although, as shown in Table 1, passive voice constructions do not play a significant role in court forms (1.2% and 1.6% of verb phrases are in passive voice), noun phrases occupy a much more predominant position (17.9% and 18.3%) and tend to be the longest constructions within sentences (e.g. Example 1 below).

Because longer sentences mostly appear in explanatory sections which present crucial procedural and legal information for filling in court forms, such as defining legal terms or legal scope (see Example 1–3 below), they impact the overall comprehension of court forms. The following examples illustrate the challenges long syntactic constructions present; their analysis is followed by a reflection on the rationale for their length and potential for enhancing their comprehensibility. For instance, the longest sentence in the most frequent forms sub-corpus appears in the guidance document “Notes to help you fill in form C1 Confirmation Inventory and form C5 (2006), HM Revenue & Customs Return”, designed to support the completion of the C1 and C5 forms. The sentence in Example 1 is the only guidance provided for the question category which says “Net qualifying value of estate”:

### **Example 1**

(82 words): *‘To work out the amount of spouse or civil partner, or charity exemption for the purposes of the excepted estates regulations, where there are people entitled to claim legitim, you will have to work out the amount of the legitim fund and then adjust the amount which would be payable to the spouse or civil partner or charity if the legitim fund were claimed in full after taking account of any legitim claimed or renounced before the application for Confirmation is made.’*

The sentence relates to working out the gross value of an estate for inheritance tax purposes. Aside from referring to specialist legal and financial terms (*legitim fund*) and legal processes (*claimed or renounced*), the sentence identifies legal scope by including the range of possible participants (*spouse, civil partner, or charity*) and establishing possible conditions and procedures (*where there are people entitled to claim legitim*, *if the legitim fund were claimed in full*). The identification of legal scope and the intention to provide an accurate description break the linear flow of information (*to work out the amount…, you will have to work the amount of the legitim fund and then adjust the amount…*). For instance, the adverbial clause of reason (*where there are people entitled to claim legitim*) incorporates explicit specification of the identity of referent [15] and breaks the infinitive clause from the main clause. Another series of coherence challenges is introduced through the co-ordination of the main clause (*you will have to work out … and then adjust…*), the conditional sentence used for hypothetical situations (*would be payable … if the legitim fund were claimed)*, ambiguous language (*adjust the amount*), unclear time-reference due to the syntactic position of the embedded clauses (the adverbial clause *after taking account of any legitim claimed or renounced* may relate to the second main clause, *adjust the amount which would be payable,* or the conditional clause *if the legitim fund were claimed*; the adverbial clause *before the application for Confirmation is made* may equally relate to the second main clause, *adjust the amount which would be payable,* or the adverbial clause *after taking account of any legitim claimed or renounced*). The lexico-grammatical characteristics of the sentence are further complicated by the fact that it belongs to two highly specialised domains, law and finance embedded within the domain of estate law.

Given the conceptual complexity embedded within the sentence, a common question that arises from legal professionals is the extent to which it could be simplified linguistically [2]. Expressing the idea in several shorter sentences could help address the syntactic complexity and the broken information flow. But the lexical complexity would require framing through exemplification and a clear definition [51]. The role of examples in aiding the addresses’ comprehension has been widely recognised in educational and other professional contexts [55], but they have not been fully utilised by the legal profession. This is partly due to the fact that prototypical situations are not easily achievable for legal purposes, but also due to the resistance from the courts and judiciary against overstepping the boundaries of guidance provision and being seen as offering unsolicited legal advice [36]. Similarly, defining legal terms is a notoriously difficult task, complicated by the fact that law constantly develops and the meaning of concepts may shift due to updates to legislation or developments in case law [21]. The implicit connections between legal concepts within a system of cognate legal principles and rules, as discussed above, further complicate the comprehension of legal texts among the wider public [2: 378]. This transcends the lexico-grammatical level and formal linguistics into the cognitive and discursive domains, abounding in inherent connections. In this respect, it can be argued that the term *legitim* is more straightforward to explain than, for instance, *domestic violence*, which is loaded with cultural, historical, linguistic and legal connotations [6, 37], and the definition for which varies even among professional service providers, such as law enforcement agencies, healthcare professionals, lawyers or policy-makers [4]. The following example incorporates intertextual links, which potentially make it possible for inherent links to be expressed more explicitly. Example 3 comes from the guidance notes for the FP2 form (Application notice Part 18 of the Family Procedure Rules 2010–‘Note 3: The order you are asking the court to make’) used for adoption cases.:

### **Example 2**

(59 words in the first sentence and 79 words in the second one; 69 and 54 words without references to legislation):

*• if you are making an application under Sect. 26(3)(f) of the Adoption and Children Act 2002 (seeking an order giving permission to apply for contact with a child who an adoption agency has placed for adoption or is authorised to place for adoption), you must also attach a draft of your application form for a contact order (Form A53);*

• *if you are making an application under Sect. 42 (6) of the Adoption and Children Act 2002 (seeking an order giving permission to apply for an adoption order before the child you are intending to adopt has lived with you for the period required under the Act) you must also attach to your application notice an additional sheet giving the details required in paragraph 3.3 of the Practice Direction 18A supplementing Part 18 of the Family Procedure Rules 2010.’*

The extract refers to gap 4 in the following section of the form: ‘*I (We) [gap 1] and [gap 2] of [gap 3] intend to apply for an order (a draft of which is attached) which: [gap 4] because [gap 5]*.’ To provide the information, the applicant needs to know which order they are applying for and the only support provided is the extract in Example 2. Instead of building a coherence thread with the form and explaining the options, the guidance note focuses on the additional evidence to be provided for the two types of orders, which are presented through intertextual links. What partially supports coherence is the double identification of the orders through intertextual links to legislation and present participle clauses describing the orders (*seeking an order giving permission to apply*). A more gradual approach, first outlining the orders and only then expanding on supplemental evidence, would reduce information density and support the information flow [17]. Example 2 thus illustrates that court forms incorporate several micro genres, such as information provision micro genre resembling legislation, and elicitation micro genre, combining elements of bureaucratic and examination styles; the constant variation among the genres results in increased cognitive load and interrupts the coherence threads.

A further communicative barrier is created by the vague information in the note for gap 5: ‘*Briefly set out why you are seeking the order. Include material facts on which you rely, identifying any rule or statutory provision.’* The absence of any form of identification of specific rules or statutory provisions to follow leaves lay court users without specific information on relevant guidance. As mentioned above, it is the technical nature of legal rules and the systematicity within law that hinders comprehensibility of legal texts or legal proceedings more generally [56]. Although the gap-fill sentence in the FP2 form looks as a simple sentence, the guidance provided creates a cognitive barrier due to the increased information density (for gap 4) and lack of information (for gap 5). This results in the presentation of midinformation [53], i.e. when the information is being provided only partially or in an incomprehensible way. Informational justice (access to information about the legal process and procedures) is perceived as an important part of procedural justice [41] and any gaps in guidance materials create challenges for access to justice [16].

Overall, the exploration of long sentences has illustrated that guidance sections present crucial information, but often in an overly complex manner. Given that lexico-grammatical complexity is an inherent part of legal discourse and enables it to achieve precision, unambiguity and generalisability [12], there are some features (e.g. complexity within embedded constructions) which create unnecessary barriers. These challenges could be avoided with the introduction of information step-by-step (if necessary, according to the chronological order of procedures), dividing longer sentences into shorter information units, explaining and illustrating legal terms, and simplifying the structure of embedded clauses and phrases. One crucial aspect to explore further is noun phrases as their structural complexity is constructed through several levels of embedded phrases/clauses as part of post-modification of head nouns, which makes it difficult to unpack the propositional content (see Example 1 and the following section).

## All-Encompassing Nominalisations

Despite being recognised as a frequent feature of legal discourse, noun phrases have not been sufficiently explored from the syntactic point view within language and the law studies [31]. Bhatia [11: ch. 6] discusses three types of nominal expressions: complex nominal phrases (typically used in advertising with adjectives receding the head noun); compound nominal phrases (typically used in scientific discourse with modifying nouns preceding the head noun) and nominalisations (typically used in legislation but also in scientific writing with the head noun followed by post-modifying clauses and phrases). The above Examples 1–2 illustrate the use of multiple prepositional phrases, noun phrases, relative clauses, and -ing clauses in parentheses embedded in the top-level noun phrases. Structural complexity enables these noun phrases to express arguments in a compact way: nominalisations are essentially semantically dense units with an accurately defined referent, which enables the text to progress swiftly and create meaning through stable reference [31]. This fits well with the requirement for legal texts to be precise, unambiguous, and all-inclusive [13]. Yet, as seen in Example 1, the clarity principle can be challenged if there are too many embedded phrases/clauses within noun phrases, which makes it important to research nominalisations in court forms.

Table 2 presents an overview of the structure of noun phrases, including the types of embedded phrases/clauses. The figures presented below are impacted by the functionality of the parser: noun phrases incorporated pronouns, which resulted in a shorter average length of noun phrases and lowered the number of embedded phrases/clauses; occasional errors in noun phrases (as seen in Fig. 2) resulted in some noun phrases divided into two; the parentheticals included information in parentheses (see Example 2) but also plural forms, such as *applicant(s)*, and intra/intertextual references, such as Sect. 4(a)(b). Although these irregularities impact the findings in the table below, the focus in this section is on results drawn from the qualitative analysis of the 50 longest noun phrases, the identification of which was not impacted by the above issues.

Embedded noun phrases and prepositional phrases are the two most frequent type of structures embedded within top-level noun phrases. Similar to the discussion on the longest sentences, the top 50 longest noun phrases occur in the sections providing guidance on completing forms, thus making comprehension issues pertinent to the active engagement with the initial stages of the court process. Example 3 comes from an incomplete sentence and illustrates a phrase from Sect. 3a of the C100 form (‘Application under Sect. 8 of the Children Act 1989 for a child arrangements, prohibited steps, specific issue order or to vary or discharge or ask permission to make a Sect. 8 order’), in which the person completing the form should indicate the type of evidence they are attaching in support of domestic abuse claims. The noun phrase is one of the options listed to support the statement *‘The applicant confirms that there is evidence of domestic violence, as specified below’* (the whole section includes 20 options and 1750 words overall, such is the length and structural complexity of the Domestic Violence Evidence Sect. (3a)):

### **Example 3**

(51 words): ‘*a letter from a public authority confirming that a person with whom a prospective party is or was in a family relationship, was assessed as being, or at risk of being, a victim of domestic violence by that prospective party (or a copy of that assessment);’*

Despite the fact that it is a noun phrase, the syntax is complex due to the attempt for accuracy and all-inclusivity (covering present and past situations *‘is or was’* or real and hypothetical situations *‘being or at risk of being’*). The syntactic complexity also introduces ambiguity. For instance, it is not clear who the words ‘*person’* or ‘*prospective party’* refer to; the use of terms *applicant* and *respondent* is well-established in legal contexts, including forms, court bundles, court correspondence and guidance materials (e.g. AdviceNow guides). The ambiguous use of the two nouns (‘*person’*, ‘*prospective party’*) raises a question of whether it is beneficial to introduce lexically simplified words with the complex syntax instead of technical terms (e.g. ‘applicant’), which have a clear referent and can be explained in a transparent way. In addition to the vague terminology, there is also unnecessary verboseness and duplication (a ‘*person’* and ‘*a victim of domestic violence’*; repetition of ‘*prospective party’*, which appears as part of prepositional post-modifiers), resulting in the use of different terms for the same referent (*person, victim*). Convoluted constructions and verbose text in this instance are unnecessarily complex and make the phrase less comprehensible for court users who are not familiar with relevant procedures. Despite the widely accepted notion that it is legal terminology that makes it difficult for lay people to engage with the justice system [32], procedural understanding plays an equally important role [52, 56]. Ensuring procedures are coherently presented, for instance, by dividing them into cognitively logical steps presented chronologically (e.g. attach a copy of assessment by a public authority or a letter from a public authority confirming the assessment) plays an important part in improving the overall comprehension of the text.

**Table:** 

Number	Title
Court forms in the most frequent forms sub-corpus	
A	Notice of [intention to proceed with] a financial application to which the standard procedure applies
C1 Confirmation	C1 Confirmation Inventory Form
C79	Application related to enforcement of a child arrangements order
COP1	Court of Protection: Application form
COP3	Court of Protection: Assessment of Capacity
D8	Application for a divorce, dissolution or (judicial) separation
D36	Notice of application for decree nisi to be made absolute or conditional order to be made final
D440_818	Request for a search for a Divorce Decree Absolute at the Central Family Court
D80B	Statement in support of divorce / dissolution / (judicial) separation – unreasonable behaviour
D84	Application for a decree nisi/conditional order or (judicial) separation decree/order
E	Financial statement • For a financial order under the Matrimonial Causes Act 1973/ Civil Partnership Act 2004 • For financial relief after an overseas divorce etc. under Part 3 of the Matrimonial and Family Proceedings Act 1984/Schedule 7 to the Civil Partnership Act 2004
FEES EX50	Civil and Family Court Fees
FEES EX160	Apply for help with fees
FL401	Application for: a non-molestation order an occupation order
LOC019 Minor	Minor’s Change of Name Deed
LOC019 Adult	Change of Name Deed
N1	N1 Claim Form
N180	Directions questionnaire (Small Claims Track)
N215	Certificate of service
N225	Request for judgment and reply to admission (specified amount)
N244	Application Notice
N245	Application for suspension of a warrant and/or variation of an order
N323	Request for Warrant of Control
PA1A	Probate application—This form is for an application where the person who has died did not leave a will that deals with assets in England and Wales
PA1P	Probate application This form is for an application where the person who has died left a will
SSCS1	Notice of appeal against a decision of the Department for Work and Pensions
Guidance documents in Court forms in the most frequent forms sub-corpus	
C1 C3 (2006) Notes	Notes to help you fill in form C1 Confirmation Inventory and form C5(2006) HM Revenue & Customs Return
CB1	Making an application: Children and the family courts
CB7	Guide for separated parents: children and the family courts
N1 EasyRead Notes for Claimants	Notes to help claimants fill in a claim form
N1A	Notes for claimant on completing a claim form
N1C	Notes for defendant on replying to the claim form
N244 Notes	Application Notice (Form N244) – Notes for Guidance
SSCS1A	How to appeal against a decision made by the Department for Work and Pensions
Court forms in the child related forms sub-corpus	
A100	Consent to the placement of my child for adoption with any prospective adopters chosen by the Adoption Agency
A101	Consent to the placement of my child for adoption with identified prospective adopters
A102	Consent to the placement of my child for adoption with identified prospective adopter(s) and, if the placement breaks down, with any prospective adopter(s) chosen by the adoption agency
A103	Advance Consent to Adoption
A104	Consent to Adoption
A105	Consent to the making of an Order under Sect. 84 of the Adoption and Children Act 2002
A106	Withdrawal of Consent: Sects. 19 and 20 of the Adoption and Children Act 2002
C_PRA1	Parental Responsibility Agreement: Sect. 4(1)(b) Children Act 1989
C_PRA2	Step-Parent Parental Responsibility Agreement: Sect. 4A(1)(a) Children Act 1989
C_PRA3	Parental Responsibility Agreement: Sect. 4ZA Children Act 1989 (Acquisition of parental responsibility by second female parent)
C1	Application for an order
C1a	Allegations of harm and domestic violence (Supplemental information form)
C2	Application • For permission to start proceedings • For an order or directions in existing proceedings • To be joined as, or cease to be, a party in existing family proceedings under the Children Act 1989
C3	Application for an order authorising search for, taking charge of, and delivery of, a child
C4	Application for an order for disclosure of a child’s whereabouts
C5	Application concerning the registration of a child-minder or provider of day-care
C7	Respond to a court application about a child
C8	Confidential contact details
C9	Statement of Service
C12	Supplement for an application for a war rant to assist a person authorised by an Emergency Protection Order
C13	Supplement for an application for a Care or Supervision Order
C13A	Supplement for an application for a Special Guardianship Order
C14	Supplement for an application for authority to refuse contact with a child in care
C15	Supplement for an application for contact with a child in care
C16	Supplement for an application for a Child Assessment Order
C17	Supplement for an application for an Education Supervision Order
C17A	Supplement for an application for an extention of an Education Supervision Order
C18	Supplement for an application for a Recovery Order
C19	Application for a warrant of assistance
C20	Supplement for an application for an order to hold a child in Secure Accommodation
C23	Emergency Protection Order
C51	Application for a Parental Order: Sect. 54 or 54A of the Human Fertilisation and Embryology Act 2008
C52	Acknowledgment Sect. 54 or 54A of the Human Fertilisation and Embryology Act 2008
C60	Certificate referred to in Article 39 of Council Regulation (EC) No. 2201/2003 of 27 November 2003(1) concerning judgments on parental responsibility
C61	Certificate referred to in Article 41(1) of Council Regulation (EC) No. 2201/2003 of 27 November 2003(1) concerning judgments on rights of access
C62	Certificate referred to in Article 42(1) of Council Regulation (EC) No. 2201/2003 of 27 November 2003(1) concerning the return of the child
C63	Application: For declaration of parentage under Sect. 55A of the Family Law Act 1986
C64	Application: For declaration of legitimacy or legitimation under Sect. 56(1)(b) and (2) of the Family Law Act 1986
C65	Application: For declaration as to adoption effected overseas under Sect. 57 of the Family Law Act 1986
C66	Application for inherent jurisdiction order in relation to children: In the High Court of Justice Family Division Principal Registry/District Registry
C67	Application under the Child Abduction and Custody Act 1985 or Article 11 of Council Regulation (EC) 2201/2003
C68	Application for international transfer of jurisdiction to or from England and Wales
C69	Application for registration, recognition or non-recognition of a judgment under Council Regulation (EC) 2201/2003 or the 1996 Hague Convention
C100_0818	Application under Sect. 8 of the Children Act 1989 for a child arrangements, prohibited steps, specific issue order or to vary or discharge or ask permission to make a Sect. 8 order
C120	Witness statement template: Child arrangements—Parental dispute
C650	Application notice to vary or set aside an order in relation to children (drug and/or alcohol toxicology test after 2010)
EX80B	Legal aid/Legal Aid Agency assessment certificate in fixed fee cases
FL401A	Application for a Forced Marriage Protection Order
FL403A	Application to vary, extend or discharge a forced marriage protection order
FP2	Application notice: Part 18 of the Family Procedure Rules 2010

The clarity of noun phrases can be hindered not only by lexical complexity but also syntactic complexity and the number of embedded phrases within them. Example 4 illustrates the post-modification of the head noun in the following sentence from ‘Notes to help you fill in form C1 Confirmation Inventory and form C5(2006) HM Revenue & Customs Return’ (the noun phrase analysed is in italics):

### **Example 4**

(53 words): ‘By qualifying charity, we mean *a charity established in the European Union (or other specified country) which would qualify as a charity under the law of England and Wales, which is regulated as a charity in the country of establishment (if appropriate) and which has managers who are fit and proper persons to be managers of a charity’*.

Here it is not only the number of embedded phrases/clauses, but the hierarchical linearity within them that impedes on the comprehension. The head noun *charity* is followed by the past participle clause (starting with *established*) and two relative clauses (starting with *which is regulated* and *which has managers*); because the relative clauses refer back to the head noun, the past participle clause creates an obstacle for the linear explanation. The three conditions of a qualifying charity (qualifying as charity in England and Wales, regulated as charity in the place of establishment, appropriate management) are thus expressed in a syntactically complex manner due to the break in the linear flow of information, which does not contribute to accuracy and all-inclusiveness; the sentence even requires additional specifications in parenthesis (*or other specific countries, if appropriate*). Furthermore, the dual term *fit and proper* has an implicit legal meaning without a clear connotation in the everyday use outside of legal knowledge schema. The notable absence of specific legal points or intertextual references to legislation or further clarifications means that there is unclarity around the definition (e.g., what would make the charity qualify as a charity under the law of England and Wales, when it is appropriate for the charity to be regulated as a charity in the country of establishment, what makes managers fit and proper for managing the charity). The combination of the broken syntactic linearity of embedded phrases/clauses, missing legal specifications and overuse of arcane legal terminology make the text not only complex but also not directly useful for lay court users.

A similar type of syntactic complexity can impede comprehensibility of even less technical text. For instance, Example 5 includes a noun phrase, which is one of the options for defining a birth father from the form C51 (Application for a Parental Order (Sect. 54 Human Fertilisation and Embriology Act 2008)); although the situation described would only be on the periphery of the legal domain, it still refers to the complex real-life situation, which is reflected in the syntactic complexity:

### **Example 5**

(70 words): ‘*the man (whether or not he is the genetic father of the child) with whom a birth mother received treatment at a licensed treatment centre when he has given a notice to the responsible person stating that he consents to being treated as the father of the child and the birth mother has also given a notice that she consents to him being treated as the father of the child.*’

The post-modifying clauses identify the father through a relative clause (*with whom a birth mother received treatment…*), an adverbial clause of time (*when he has given a notice …*.) and an appended coordinate clause (*and the birth mother has also …*). The reinstatement of that fact that both parents gave their consent and the repetition of the prepositional phrase ‘*to the responsible person’* makes the text unnecessarily long and complicated as three of the embedded clauses could be shortened into one without impacting the accuracy of the statement. The unnecessary degree of complexity can be explained by the fact that the noun phrase includes both legal information (what defines the father in the human fertilisation and embryology process) and procedural information (what evidence is required for the legitimisation of parental rights); the text is thus a conglomerate of complicated legislation and a complex set of rules for legal processes and procedures.

What is shared among the noun phrases presented above (Examples, 1, 3, 4, 5) is the high degree of embeddedness within post-modifying phrases/clauses, breaks in the linear information flow, broken hierarchical organisation of embedded phrases/clauses, and unnecessary verboseness. The degree of complexity is given not only by the multi-functionality of court forms, but also by the aggregation of complex legal and procedural information presented in a discontinued manner to provide some guidance and support for the elicitation strategy embedded in the court forms.

## Summary

The study shows that there is a lot of variation among different court forms and within individual forms. The fact that court forms are so diverse is given by the wide range of legal domains they refer to, but also their multiple communicative aims and distinct genre requirements for individual sections (eliciting information, providing guidance on procedures and legal points, and giving instructions on how to complete forms). This diversity within the forms contributes to the discourse-related comprehension barriers: the links between the elicitation strategies embedded in court forms and the relevant guidance provided can create cognitive barriers (e.g. Example 2). But even on the more formal level of micro-linguistic features, the structural discontinuity within noun phrases and sentence constructions create unnecessary comprehension challenges (e.g. Examples 1, 3, 5). There is a strong argument to potentially justify complex syntax when such constructions aid accuracy of expression and support all-inclusiveness, but when they mainly introduce ambiguity and unclarity, the structural complexity is not substantiated.

This line of argumentation is further strengthened when viewed in light of procedural justice. The examples analysed here (especially the noun phrases in the examples) encompass legal content and procedural information, which means that they essentially function in the context of two broad types of legal discourses: legislation and civil/family procedure rules. Both are extremely complex from the linguistic perspective and from the point of view of complexity of systemic links within law, legislation, legal doctrines, legal processes and court procedures [2, 56]. Yet it is important to recognise that the main users of court forms are lay people and irrespective of whether they are represented, the forms need to help them present their case to the judiciary and reflect the narrative which would then be developed in court [50]. Comprehension challenges thus create barriers for accessing justice: informational justice and access to transparent information is part of procedural justice [16, 41].

Court forms are a typical example of the problem-solving type of legal setting because they relate to a specific legal problem the lay user is facing [56]. Court users thus have to engage with the court process in the reactive manner (where the opportunity for planning is minimal as they need to submit an application after the problem occurred) and the court form creates a starting point for identifying relevant legal texts within the context of the problem. The process of completing the form is part of the legal hermeneutics, which involves not only “understanding the text, but construing, formulating and interpreting the factual situation” [56: 257]. The extracts analysed above offer some support with the identification of the relevant court processes or legislation, but they also illustrate gaps in coherence links, missing information, and information presented in unnecessarily complex manner. This leads to the institutional position of providing mid-information [53], reinforcing the unequal status of the lay court user [18: 162–169] and increasing the potential for common misconceptions about law [30]. And although easified legal texts [7] are not easily achievable, and full comprehension of legal discourse by the lay person is possibly unachievable at all [2], information equity is still crucial for the active engagement with the proceedings. There needs to be, however, a wider recognition among legal professionals of the importance of embedding clear and coherent legal and procedural information into court forms [36]. It then becomes a question of drafting with the appropriate attention to audience design and focus on the lay reader; comprehensibility optimisation should be an inherent part of the drafting process from the initial stages rather than part of a follow-up reformulation process [54].

The study shows that court forms as a tool for legal-lay communication need to be further explored from the linguistic and socio-legal perspectives. The linguistic features which would contribute to improving their comprehensibility range from micro-linguistic features (e.g. structurally transparent sentences and noun phrases, clearly defined terminology, illustrations of complex situations through examples or visualisations) to discursive characteristics (e.g. coherence links between questions and guidance, contextualising of questions within relevant legal concepts and principles and procedural requirements). The comprehensibility of court forms, or the application process in general, will become even more pertinent to the digitised proceedings or any form of AI assistance as part of the legal dispute resolution process because such processes equally rely on the need to elicit information from the lay person: the comprehensibility of elicitation strategies and guidance materials should thus be an inherent part of the (computer-assisted) legal-lay interaction.

## Data Availability

All court forms were collected from https://www.gov.uk/search/all. The Appendix lists the forms which were collected for the analysis.

## Footnotes

1 Courts only collect numbers of self-represented parties in private family hearings, which makes it difficult to estimate the number of self-represented parties in other types of proceedings. https://www.gov.uk/government/statistics/family-court-statistics-quarterly-april-to-june-2021.

2 https://insidehmcts.blog.gov.uk/2021/06/09/remote-hearings-their-role-in-extending-access-to-justice/.

3 https://assets.publishing.service.gov.uk/government/uploads/system/uploads/attachment_data/file/885814/form-a-eng.pdf.

4 The list of all court forms in both sub-corpora can be found in the Appendix. The form C100 was include in both sub-corpora because it is one of the most frequently downloaded forms and also the form used for child arrangement orders.

5 https://www.sketchengine.eu/.

6 https://basex.org/.

## Code Availability

All software used for the analysis is cited in the article.
